# Rejuvenating the Aging Heart by Enhancing the Expression of the *Cisd2* Prolongevity Gene

**DOI:** 10.3390/ijms222111487

**Published:** 2021-10-25

**Authors:** Chi-Hsiao Yeh, Yi-Ju Chou, Ting-Kuan Chu, Ting-Fen Tsai

**Affiliations:** 1 College of Medicine, Chang Gung University, Taoyuan 333, Taiwan; yehccl@cgmh.org.tw; 2 Department of Thoracic and Cardiovascular Surgery, Chang Gung Memorial Hospital, Linkou, Taoyuan 333, Taiwan; 3 Institute of Molecular and Genomic Medicine, National Health Research Institute, Zhunan, Miaoli 350, Taiwan; yjchou0810@nhri.edu.tw; 4 Department of Life Sciences and Institute of Genome Sciences, National Yang Ming Chiao Tung University, Taipei 112, Taiwan; a00606457@gmail.com; 5 Aging and Health Research Center, National Yang Ming Chiao Tung University, Taipei 112, Taiwan

**Keywords:** cardiac aging, cardiac rejuvenation, *Cisd2*, inducible cardiac-specific overexpression

## Abstract

Aging is the major risk factor for cardiovascular disease, which is the leading cause of mortality worldwide among aging populations. *Cisd2* is a prolongevity gene that mediates lifespan in mammals. Previously, our investigations revealed that a persistently high level of *Cisd2* expression in mice is able to prevent age-associated cardiac dysfunction. This study was designed to apply a genetic approach that induces cardiac-specific *Cisd2* overexpression (*Cisd2* icOE) at a late-life stage, namely a time point immediately preceding the onset of old age, and evaluate the translational potential of this approach. Several discoveries are pinpointed. Firstly, *Cisd2* is downregulated in the aging heart. This decrease in *Cisd2* leads to cardiac dysfunction and impairs electromechanical performance. Intriguingly, *Cisd2* icOE prevents an exacerbation of age-associated electromechanical dysfunction. Secondly, *Cisd2* icOE ameliorates cardiac fibrosis and improves the integrity of the intercalated discs, thereby reversing various structural abnormalities. Finally, *Cisd2* icOE reverses the transcriptomic profile of the aging heart, changing it from an older-age pattern to a younger pattern. Intriguingly, *Cisd2* icOE modulates a number of aging-related pathways, namely the sirtuin signaling, autophagy, and senescence pathways, to bring about rejuvenation of the heart as it enters old age. Our findings highlight *Cisd2* as a novel molecular target for developing therapies targeting cardiac aging.

## 1. Introduction

Aging is the major risk factor for the development of multi-morbidity-related age-associated disorders such as cardiovascular diseases, various cancers, diabetes, and a range of neurodegenerative diseases [1]. Notably, cardiovascular disease is the leading cause of mortality among persons aged over 65 years [2]. Approximately one third of all deaths in older persons are due to heart disease [3]. However, research has shown that among Americans younger than 80 years, 15–43% of the deaths caused by cardiac diseases are preventable via appropriate interventions and/or a modification of lifestyle [4]. During cardiac aging, non-proliferative fully differentiated cardiomyocytes are affected by a variety of problems that gradually bring about structural damage and functional decline [5]. Previous studies have revealed that cumulative oxidative stress results in an accumulation of damaged proteins, of dysfunctional organelles, and of DNA damage. In addition, these alterations also play a detrimental role that results in the aging heart suffering from cellular senescence and functional decline.

Using murine models, an increased accumulation of senescent cardiomyocytes has been found as early as at 12 months of age, namely at a murine middle age. One of the characteristics of cardiac aging is its effect on the tolerance of stress. The stress tolerance of the heart begins to decrease at 18 months of age. Subsequent to this, there is a decrease in cardiac function; this occurs six months after the detection of senescent cardiomyocytes [6]. Interestingly, cardiomyocyte senescence can be alleviated by preventing mitochondrial dysfunction [7,8] or by the elimination of damaged cell components via upregulation of autophagy [9,10]. Mitochondrial damage is often found in aged hearts and this includes disorganized organelles, a loss of mitochondrial inner membrane cristae, and enlargement of the distance between the mitochondria and other organelles [11]. Age-related mitochondrial dysfunction is also evident in the aged heart at the functional level. This includes decreased ATP production, increased ROS generation, dysregulation of Ca^2+^ homeostasis, and defective quality control. In addition, deficiencies in autophagy and in the lysosome-dependent degradation pathways also play a crucial role in the development of dysfunctional organelles; these contain damaged molecules that hinder the recycling of cellular components in the aged heart [12]. All of these age-related defects contribute to the structural damage and functional decline within the aging heart and eventually result in heart failure [13]. As the field has advanced, treatments targeting the alleviation of age-related cardiac disorders appear to be inextricably linked to therapeutics that target the mitochondrion and the biological processes related to the mitochondrion.

*Cisd2* is an evolutionally conserved gene that plays an essential role in mediating mammalian lifespan [14]. The *Cisd2* protein is mainly located in the mitochondrial outer membrane, the endoplasmic reticulum (ER), and the mitochondria-associated membranes (MAMs). During the natural aging of wild-type mice, the level of expression of *Cisd2* decreases in a range of organs and tissues, including skeletal muscles, liver, brain, and skin [15,16,17,18,19,20,21]. Importantly it has been noted that *Cisd2* deficiency leads to mitochondrial dysfunction, disruption of cytosolic Ca^2+^ homeostasis, elevation of ROS production, and dysregulation of autophagy. These defects in the *Cisd2* knockout (*Cisd2*KO) mice are linked to a shortened lifespan and a premature aging phenotype [15]. Conversely, a persistently high level of *Cisd2* expression in *Cisd2* transgenic (*Cisd2*TG) mice extends their median and maximum lifespan without any apparent deleterious side effects [22].

Specifically, in the heart, our previous investigations revealed that the decreased cardiac level of *Cisd2* that occurs during aging results in disturbances to electrical conductance and mechanical contraction dysfunction [17]. Intriguingly, our previous results also demonstrated that maintaining the *Cisd2* protein at a persistently high level, namely one comparable to the *Cisd2* level at a young age, can delay cardiac aging and attenuate the age-related functional decline of the heart; this is what occurs in *Cisd2*TG mice that carry two additional copies of the *Cisd2* gene [17]. However, it remains unclear as to whether the anti-aging effect of *Cisd2* on the heart is a direct effect of *Cisd2* on cardiomyocytes. Thus, does the *Cisd2* function during cardiac aging act in an autonomous manner, and/or does it act indirectly via crosstalk between the heart and other organs/tissues? This study was designed to apply a genetic approach that enhances the expression level of *Cisd2* in a spatial-specific and temporal-specific manner, namely in the aging heart at a late-life stage (18 months). Intriguingly, our findings reveal that after *Cisd2* is downregulated at 18 months of age, an induction of *Cisd2* overexpression is able to indeed reverse cardiac aging and rejuvenate the structure and functioning of the aging heart.

## 2. Results

### 2.1. Enhancing Cisd2 in Aging Hearts via Inducible Cardiac-Specific Cisd2 Overexpression (Cisd2 icOE)

**Figure 1:**
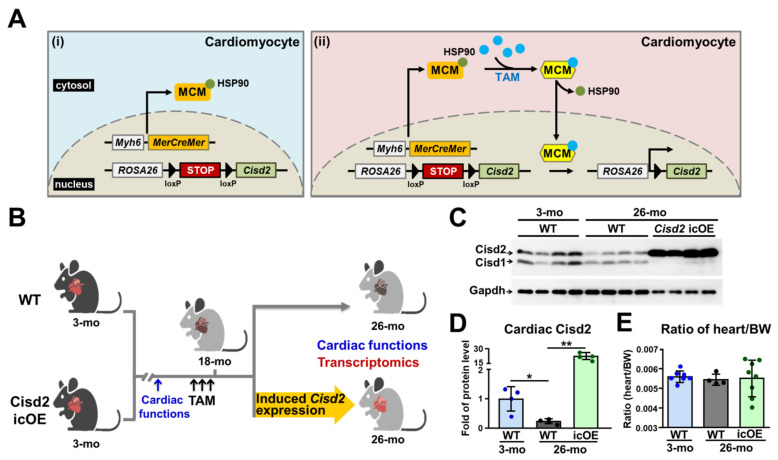
Schematic illustration of the inducible and cardiac-specific overexpression of *Cisd2* (*Cisd2* icOE) in transgenic mice. (**A**) The strategy used for cardiac-specific and tamoxifen (TAM)-inducible overexpression of *Cisd2* in the mice. The *R26-Cisd2KI/+* mice were crossed with mice carrying the cardiac-specific expression of tamoxifen-inducible Cre recombinase (*αMHC-MerCreMer*). (i) In the absence of tamoxifen, the MerCreMer (MCM) is sequestered by heat shock protein 90 (HSP90) and retained in the cytoplasm. (ii) Under the presence of tamoxifen, the MCM complex is disassociated from HSP90 and is then translocated into the nucleus to carry out the Cre/loxP-mediated deletion of the STOP cassette; *αMHC*, *α-myosin heavy chain* promoter. (**B**) The flow chart of tamoxifen treatment. The cardiac functions were monitored at 3 and 18 months of age before tamoxifen injection. At 18 months of age, both the WT control (*R26-Cisd2KI/+*) and the *Cisd2* icOE (*R26-Cisd2KI/+; αMHC-MCM*) mice had tamoxifen administered by intraperitoneal injection (40 mg/kg body weight on three consecutive days). Cardiac function analysis was performed at 26 months of age, namely 8 months after tamoxifen injection. (**C**,**D**) Western blot analysis and quantification of the expression levels of *Cisd2* in the hearts obtained from 3-mo WT, 26-mo WT, and 26-mo *Cisd2* icOE mice. (**E**) Ratio of heart weight and body weight (BW). The data are presented as the means ± SD and analyzed by one-way or two-way ANOVA with Bonferroni multiple comparison tests; * indicates *p* < 0.05; ** *p* < 0.005. There were at least five mice in each group. The figure was created with BioRender.com.

Our previous study demonstrated that the level of *Cisd2* expression in the heart is decreased in mice after the age of 13 months [14], and the *Cisd2* protein level in the myocardium of 26-mo wild type (WT) mice is significantly reduced by about 50% compared to 3-mo WT mice [17]. We also showed that a reduced level of *Cisd2* is associated with age-related cardiac dysfunction [17]. To evaluate the translational potential of *Cisd2* in terms of its therapeutic effect on age-associated cardiac dysfunction, we generated a murine model that carries a conditional *Cisd2* transgene located in the ROSA26 locus and is driven by the endogenous *ROSA26* promoter (*R26-Cisd2KI/+*; Supplementary Figure S1). The *R26-Cisd2KI/+* mice were crossed with *αMHC-MerCreMer* (MCM) transgenic mice, which carry the inducible and cardiac-specific MerCreMer (Figure 1A). Tamoxifen-inducible Cre recombinase, namely MerCreMer, is a fusion protein that contains Cre recombinase with two modified estrogen receptor ligand-binding domains at either end [23]. Overexpression of *Cisd2* was induced at 18 months of age (Figure 1B). This age in mice is equivalent to the late stage of human middle age that immediately precedes the onset of human old age [24]; furthermore, it should be noted that there is a significant increase in the prevalence of congestive heart failure in humans at a late middle age [25]. Consistently with our previous results, it was found that the *Cisd2* protein level in the heart of the 26-mo WT control (*R26-Cisd2KI/+*) mice is significantly decreased; on the other hand, the level in the *Cisd2* icOE (*R26-Cisd2KI/+; αMHC-MCM*) mice was found to be significantly increased after tamoxifen injection from the age of 18 months (Figure 1C,D). The ratio of heart weight to body weight of the WT and *Cisd2* icOE mice at 26 months of age showed no obvious difference (Figure 1E).

### 2.2. Cisd2 icOE Prevents an Exacerbation of the Age-Associated Electromechanical Dysfunction

**Figure 2:**
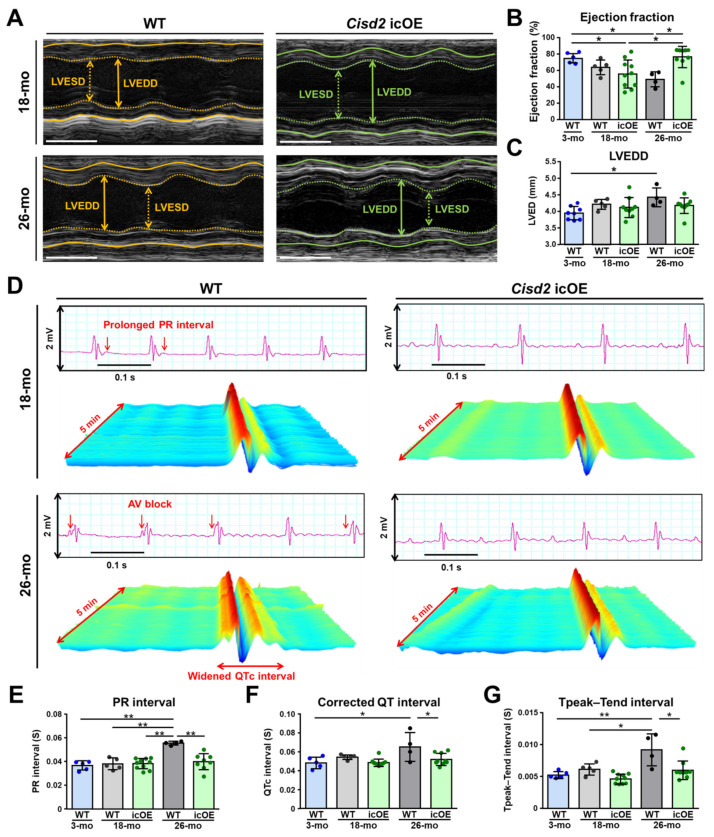
Cardiac-specific overexpression of *Cisd2* at a late-life stage rescues age-related electromechanical dysfunction of the aging heart. (**A**) Representative echocardiographs obtained from the WT control (*R26-Cisd2KI/+*) mice and the *Cisd2* icOE (*R26-Cisd2KI/+; αMHC-MCM*) mice (before 18 months of age) and after tamoxifen injection for 8 months (26 months). White bar: 0.1 s. Echocardiographs of the 3-mo WT mice were used as the control. (**B**,**C**) Left ventricular ejection fraction (EF) and end-diastolic diameter were measured and quantified by echocardiography. Left ventricular EF decreased in the WT (*R26; Cisd2KI/+*) mice during aging. However, in the *Cisd2* icOE mice, their EF had increased at 26 months of age after induction of *Cisd2* overexpression for 8 months compared with that at 18 months. In the WT mice, the LVEDD significantly increased from the age of 18 months to the age of 26 months. However, in the *Cisd2* icOE mice, the LVEDD did not increase at 26 months of age compared with 18 months of age. (**D**) Representative waterfall plots and ECG tracings recorded following anesthesia of the mice. Atrioventricular (AV) block with irregular and progressive prolongation of the PR interval (**E**), a widened corrected QT (QTc) interval (**F**), and a prolonged Tpeak–Tend interval (**G**) were found in the WT mice at 26 months of age. However, cardiac-specific overexpression of *Cisd2* appears to have reversed the changes in cardiac function and prevented the heart from suffering from disturbances in electrical conduction. The data are presented as the means ± SD and are analyzed by one-way or two-way ANOVA with Bonferroni multiple comparison tests; * *p* < 0.05; ** *p* < 0.005. There were at least five mice in each group. LVEDD, left ventricular end-diastolic diameter; LVESD, left ventricular end-systolic diameter.

In 18-mo WT mice, echocardiography revealed that their hearts displayed a variety of left ventricular contractile dysfunctions (Figure 2A,B). As the age of the mice advanced from 18 months to 26 months, there was a progressive and significant reduction in the left ventricular (LV) ejection fraction of the naturally aged mice. However, when the *Cisd2* icOE mice were examined, the trajectory of LV ejection fraction reduction was reversed after cardiac-specific *Cisd2* overexpression at the age of 18 months. Specifically, the LV ejection fraction of the 26-mo *Cisd2* icOE mice was significantly elevated after cardiac-specific *Cisd2* overexpression. Additionally, age-associated dilation of their cardiac chambers was found in the 26-mo WT mice, which were found to be significantly larger than at 18 months (Figure 2C). Thus, cardiac-specific *Cisd2* overexpression was found to prevent age-associated dilatation of cardiac chamber. Moreover, in the old WT mice, a longitudinal analysis of their electrocardiography (ECG, Figure 2D) revealed that during natural aging, there were progressive prolongations of the PR interval (Figure 2E), the corrected QT interval (QTc, Figure 2F), and the Tpeak–Tend interval (Figure 2G) as the age of the mice increased from 18 months to 26 months. Remarkably, in the 26-mo *Cisd2* icOE mice, the PR interval, the QTc interval, and the Tpeak–Tend interval were similar to those of the 18-mo mice. Taken together, these echocardiography and ECG findings reveal that the age-associated decrease in the level of cardiac *Cisd2* appears to bring about electromechanical dysfunction in the murine heart. Furthermore, and intriguingly, cardiac-specific induction of *Cisd2* overexpression at a late-life stage, namely at 18 months of age, can prevent the exacerbation of these disturbances in heart electric conductance associated with aging and it also reverses the decline in contractile capability linked to natural aging.

### 2.3. Cisd2 icOE Reverses Age-Associated Cardiac Fibrosis and Intercalated Disc Defects

**Figure 3:**
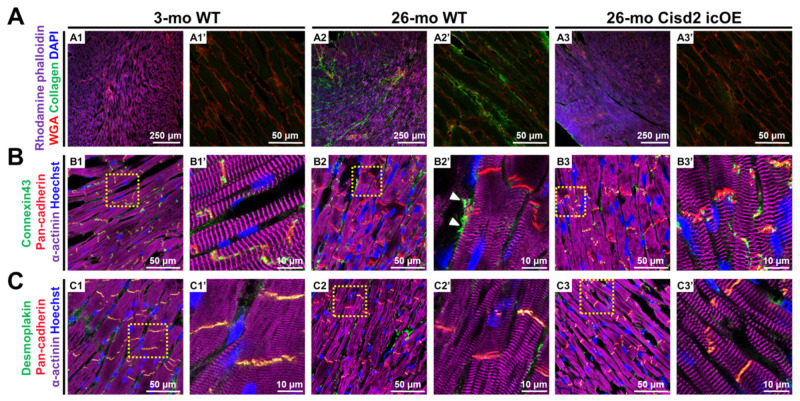
Cardiac-specific overexpression of *Cisd2* at a late-life stage rescues the aging hearts of mice from age-related cardiac fibrosis and intercalated disc (ICD) defects. (**A**) Age-related cardiac fibrosis can be seen to be ameliorated by enhancing the expression level of *Cisd2* in the aging heart. Representative immunofluorescence images of heart sections from the three groups of mice (3-mo WT, 26-mo WT, and 26-mo *Cisd2* icOE) stained with antibodies against collagen I (green) and wheat germ agglutinin (red) to identify the cell membrane, with rhodamine phalloidin—to identify actin (purple), and with DAPI—to identify the cardiomyocytic nucleus. Age-related cardiac fibrosis is obvious in the 26-mo WT mice (**A2**). However, overexpression of cardiac *Cisd2* appears to reduce cardiac fibrosis (**A3**). (**B**,**C**) Overexpression of *Cisd2* reverses the age-related defects affecting the ICD in the aging heart. Representative immunofluorescence images of heart sections from the three groups of mice (3-mo WT, 26-mo WT, and 26-mo *Cisd2* icOE) stained with antibodies against Cx43 (green) was used to localize gap junctions, against pan-cadherin (red)—to localize the ICDs, and against α-actinin (purple)—to stain muscle fibers. The sections were also stained with Hoechst (blue) to identify the nuclei. Cx43 is well-co-localized with pan-cadherin in hearts of the 3-mo WT mice (**B1**). However, lateralization and loss of co-localization of Cx43 with pan-cadherin were found in hearts of the 26-mo WT mice (**B2**). White arrows indicate lateralization of gap junctions. Maldistribution of desmosomes was found in the hearts of 26-mo WT mice (**C**). Co-localization of desmoplakin (green) with pan-cadherin (**C1**) was found within desmosomes of the 3-mo WT mice, and this was lost in hearts of the 26-mo WT mice (**C2**). Remarkably, defects in the ICD were reversed by cardiac-specific overexpression of *Cisd2* (**B3**,**C3**).

To define the structural basis of age-associated cardiac dysfunctions brought about by a decline in the *Cisd2* protein level, we performed IF staining to examine the cardiac interstitial fibrosis and the integrity of the components of the intercalated discs (ICDs), specifically the gap junctions and desmosomes. Notably, interstitial fibrosis was evident in the hearts of 26-mo WT mice. Intriguingly, in the 26-mo *Cisd2* icOE mice, cardiac-specific *Cisd2* overexpression significantly ameliorated the presence of interstitial fibrosis (Figure 3A). Furthermore, IF staining of connexin 43 (Cx43, a gap junction protein) revealed that in the 26-mo WT mice, the age-associated decrease in the *Cisd2* protein appears to cause amplification and lateralization of the gap junctions (Figure 3B). In the 26-mo WT mice, IF staining of the Cx43 was observed along the lateral borders of the cardiomyocytes; lateralization of the gap junctions has been shown to be associated with conductance disturbance during aging [26]. Furthermore, the co-localization coefficient of Cx43 and pan-cadherin was significantly decreased in the naturally aged hearts of the 26-mo mice, which suggests that a significant portion of the lateralized gap junctions were not co-localized with ICDs (Supplementary Figure S2). Moreover, in the 26-mo WT mice, maldistribution of desmosomes was detected by IF staining of desmoplakin (Figure 3C). During cardiac contraction, desmosomes play an important role because they help to link all the cardiomyocytes via the binding of the intermediate filaments present in the heart. Accordingly, maldistribution of desmosomes along with the interstitial fibrosis of collagen deposition contribute to age-associated contractile dysfunction. Remarkably, in the *Cisd2* icOE mice, the lateralization of gap junctions was significantly decreased, while the co-localization coefficient of Cx43 and pan-cadherin was significantly increased (Figure 3, Supplementary Figure S2). Taken together, these results show us that induction of cardiac-specific *Cisd2* overexpression at a late-life stage can improve the integrity of the ICDs and also ameliorates cardiac fibrosis: thus, there is a reversal of the structural abnormalities normally present in an aging heart.

### 2.4. Cisd2 icOE Changes the Cardiac Transcriptomic Profile from an Old-Age Pattern to a Younger Pattern

**Figure 4:**
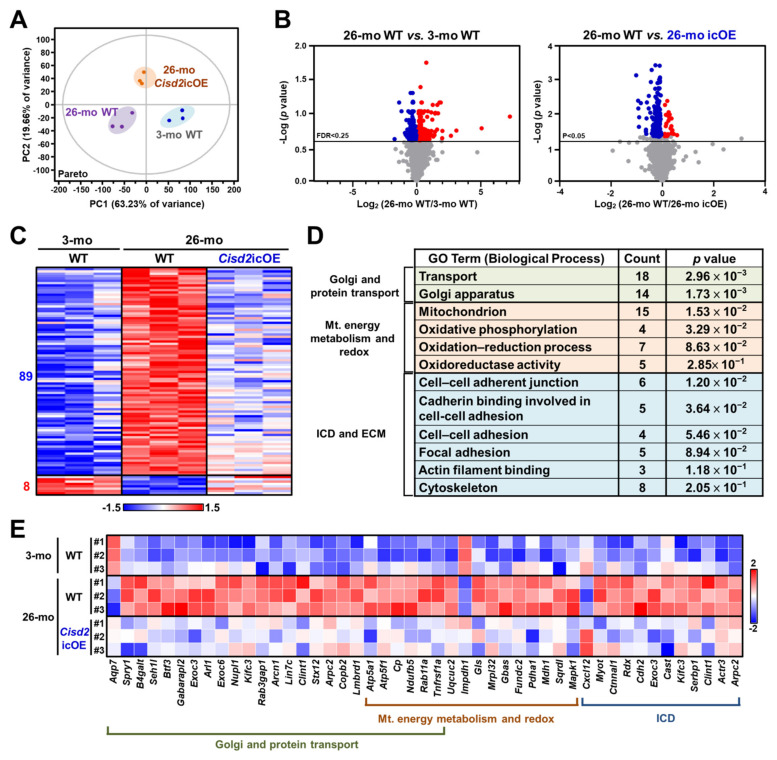
Cardiac-specific overexpression of *Cisd2* at late-life stage in *Cisd2* icOE mice reverses the cardiac transcriptomic profile changing it from an old-age pattern to a younger pattern. (**A**) Principal component analysis (PCA) of the RNA-seq data from the three groups of mice, namely 3-mo WT, 26-mo WT, and 26-mo *Cisd2* icOE. (**B**) Volcano plots representing the transcriptomic changes in the 26-mo WT vs. 3-mo WT mice and in 26-mo WT vs. 26-mo *Cisd2* icOE mice. The horizontal lines show the 25% false discovery rate (FDR) or the *p* < 0.05 threshold. Blue dots indicate the downregulated genes and red dots indicate the upregulated genes. (**C**) Heatmap showing the 97 differently expressed genes (DEGs) that are reverted by the cardiac-specific overexpression of *Cisd2* in the *Cisd2* icOE mice. (**D**) Analysis of the *Cisd2*-reverted DEGs by the GO Biological Processes. (**E**) Heatmap showing the DEGs involved in Golgi and protein transport, mitochondrial (Mt.) energy metabolism and redox, and ICDs and ECM. ICD, intercalated disc; ECM, extracellular matrix.

To identify the molecular basis of *Cisd2*-mediated rejuvenation in the aging heart, the transcriptomic profiles of hearts were generated using the RNA sequencing (RNA-seq) technology. We carried out the following comparisons: (a) 26-mo WT vs. 3-mo WT mice (natural aging) and (b) 26-mo *Cisd2* icOE vs. 26-mo WT mice (*Cisd2*-mediated rejuvenation). The expression levels of a total of 5686 genes were quantified during this part of the study. The transcriptomic profiles of old mice (26-mo) and young mice (3-mo) showed a dramatic difference when principal component analysis (PCA) was carried out. Notably, the transcriptomic profile of the *Cisd2* icOE (26-mo) mice was more clustered together and closer to that of the young WT (3-mo) mice. This indicates that overexpression of *Cisd2* in the aging heart during late life has an influence on the cardiac transcriptome profile (Figure 4A). A total of 866 differentially expressed genes (DEGs), namely those genes that were either upregulated or downregulated, were found in the naturally aged hearts (26-mo) compared to the young hearts (3-mo). Remarkably, 97 DEGs were reverted by cardiac-specific overexpression of *Cisd2*. Among these 97 *Cisd2*-reverted DEGs, 89 genes were found to be initially upregulated and eight genes were found to be initially downregulated in the naturally aged hearts (Figure 4B,C). Furthermore, gene annotation analysis of biological processes by Gene Ontology (GO) associated with DEGs revealed that the *Cisd2*-reverted DEGs can be mainly classified into three functional groups: (a) Golgi and protein transport, (b) mitochondrial energy metabolism and redox, and (c) the intercalated discs and extracellular matrix (Figure 4D,E). Interestingly, these *Cisd2*-mediated changes in the biological processes are consistent with previous findings on the functional and histological alterations present in aged hearts (Figure 2 and Figure 3).

### 2.5. Analyses of Biological Functions and Pathways Associated with the DEGs Reverted by *Cisd2* icOE

**Figure 5:**
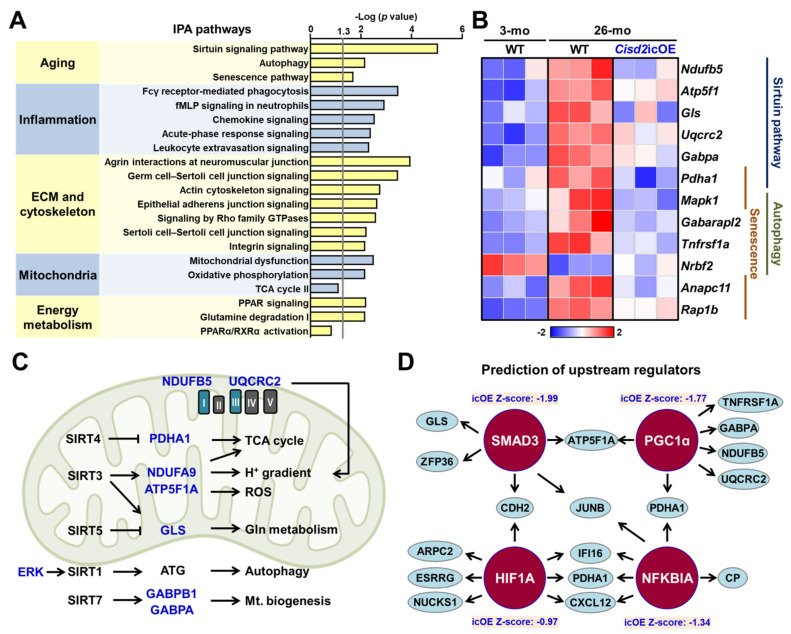
Analyses of the biological functions and pathways of the DEGs reverted by cardiac-specific *Cisd2* overexpression. (**A**) Ingenuity pathway analysis (IPA) of the DEGs. (**B**) Heatmaps representing the *Cisd2*-reverted DEGs (26-mo *Cisd2* icOE vs. 26-mo WT mice, *p* < 0.05) related to the sirtuin, autophagy, and senescence pathways. (**C**) The diagram illustrates the relationships between the SIRT family genes and their related biological functions and the *Cisd2*-reverted genes. The significant genes reverted by *Cisd2* are highlighted with blue color (*p* < 0.05). (**D**) The upstream regulators obtained by IPA upstream regulator analysis. The figure was created with BioRender.com.

In order to obtain insights into the molecular mechanisms by which *Cisd2* icOE rejuvenates the aging heart, we carried out an ingenuity pathway analysis (IPA) using the following comparisons: (a) 26-mo WT vs. 3-mo WT mice (natural aging) and (b) 26-mo *Cisd2* icOE vs. 26-mo WT mice (*Cisd2*-mediated rejuvenation). Among the biological processes and/or pathways identified, the top five items were found to be (a) aging, (b) inflammation, (c) the extracellular matrix and the cytoskeleton, (d) mitochondria, and (e) energy metabolism (Figure 5A). Importantly, all of these are highly associated with the structural alterations and functional decline that occurs during cardiac aging. Specifically, the DEGs involved in a number of aging-related pathways, namely the sirtuin, autophagy, and senescence pathways, are known to be dysregulated in the aging heart. However, cardiac-specific *Cisd2* overexpression does appear to reverse the dysregulation of these DEGs and move the expression pattern towards the younger one observed in the 3-mo mice (Figure 5B). An overview of the interconnections between the three pathways, namely the sirtuin, autophagy, and senescence pathways, is shown in Figure 5C. Moreover, using IPA upstream regulator analysis, we were able to predict the upstream regulators that are affected by *Cisd2* overexpression. We found the following four transcription factors to be upregulated during cardiac aging; importantly, these were found to be downregulated by *Cisd2* overexpression (*z*-score < 0). They are (a) *SMAD family member 3* (*SMAD3*), (b) *peroxisome proliferator-activated receptor gamma coactivator 1 alpha* (*PGC1-α*), (c) *hypoxia-inducible factor 1 alpha* (*HIF1α*), and (d) *NFKB inhibitor alpha* (*NFKBIA*) (Figure 5D). Taken together, these findings indicate that the induction of cardiac-specific *Cisd2* overexpression can reverse aspects of the cardiac transcriptomic profile from an old-age pattern to a younger pattern and thereby rejuvenate the aging heart.

## 3. Discussion

In this study, we provided genetic evidence to substantiate the hypothesis that an age-associated decrease of *Cisd2* causes electromechanical dysfunction in the heart. Furthermore, we also showed that cardiac-specific overexpression of *Cisd2* can rejuvenate the aging heart. Several findings can be pinpointed. Firstly, *Cisd2* is downregulated in the aging heart. This decrease in *Cisd2* leads to cardiac dysfunction and impairs electromechanical performance. Intriguingly, *Cisd2* icOE at a late-life stage, namely a timepoint that immediately precedes the onset of old age, prevents the exacerbation of this age-associated electromechanical dysfunctionality. Secondly, *Cisd2* icOE results in amelioration of cardiac fibrosis and improves the integrity of ICD, thereby largely reversing the structural abnormalities present in the aging heart. Maintaining the integrity of the ICD is crucial to heart functionality as this allows the rapid spread of electric impulses and the synchronization of all cardiomyocytes, which allows them to contract as a single functional organ. Finally, *Cisd2* icOE reverses the transcriptomic profile of the aging heart, changing it from an old-age pattern to a younger pattern. Intriguingly, during this process, the *Cisd2* icOE state modulates various aging-related pathways, specifically the sirtuin signaling, autophagy, and senescence pathways; these changes bring about rejuvenation of the heart at an old age.

### 3.1. Current Strategies for Heart Rejuvenation and Their Limitations

#### 3.1.1. Exercising at an Old Age

Many studies have demonstrated that exercising initiated at an age when deleterious alterations in the heart have already begun can attenuate cardiac fibrosis and collagen crosslinking that occur when an individual reaches an advanced age; these are important contributors to morbidity and mortality at an old age [27,28]. A human study also demonstrated that lifelong endurance training can preserve cardiac function at a level similar to that of young subjects, although the mechanism remains unknown [29]. One of our previous studies provided evidence that exercising induces *Cisd2* expression, which improves mitochondrial function and restores a younger metabolic profile [28]. Therefore, it seems quite possible that exercising rejuvenates the aging heart via an upregulation of *Cisd2* expression.

#### 3.1.2. Cell Therapy

In contrast to preclinical claims of structural and functional recovery after myocardial infarction (MI), cell therapy, when used with acute and chronic MI models, has been largely disappointing [30,31,32]. The results of clinical trials have failed to verify that cell therapy is able to restore heart function after injury [32]. Cardiac fibrosis has been recognized as the fundamental response of the heart to various injuries, including but not limited to inflammation, ischemia/reperfusion, and infection. Previous studies revealed that stress-induced increase in proinflammatory cytokines and profibrotic factors resulted in the proliferation of cardiac fibroblasts accompanied with elevated secretion of collagens and other extracellular matrix proteins. Subsequently, the accumulation of excessive extracellular matrix proteins led to cardiac remodeling with architecture distortion and cardiac dysfunction [33]. A recent alternative approach has been the development of cell therapy targeting cardiac fibrosis; this involves the adoptive transfer of T cells that target the fibroblast activation protein. This approach has been found to result in a significant reduction in cardiac fibrosis and restoration of cardiac function [34]. Senescent cells, which accumulate during aging, are known to disrupt cardiac structure and function [35] and represent another therapeutic target when preventing age-associated cardiac dysfunction [6]. Clearance of cardiac senescent cells has been shown to delay aging-associated cardiac dysfunction [35] and extend the healthy lifespan [6]. Another discovery related to cardiac health is that mesenchymal stem cells are able to transfer mitochondria through nanotubular structures to cells that have mitochondria with defective mDNA; this shed light on a possible therapeutic role for mitochondrial transplantation [36]. Mitochondrial transplantation has also been proposed as a means of cardiac rejuvenation, especially when stem cell-derived mitochondria are used [37]. Young healthy mitochondria, when obtained from stem cells, have a better mitochondrial membrane potential and a higher transcription rate, and thus are a good resource of donor mitochondria [38]. Evidence has also been found proving that a reduction in autophagy and mitophagy brings about acceleration of the aging process, while on the other hand the enhancement of these processes preserves cardiac homeostasis and extends the lifespan [39]. Our previous studies have demonstrated that overexpression of *Cisd2* preserves cardiac mitochondrial function and reduces reactive oxygen species production, thus attenuating age-associated cardiac dysfunction [11,17]. The structure of mitochondria and the interactions that occur between mitochondria and other organelles are also well-preserved when there is an elevation of *Cisd2* expression. This strongly suggests that *Cisd2* has great potential as a treatment target for cardiac rejuvenation.

#### 3.1.3. Calorie Restriction

Hitherto, calorie restriction (CR) has been considered to be the most effective intervention for delaying aging and age-associated diseases across a wide variety of organisms [40,41]. Initiation of CR at a middle or old age, but not at a young age, is believed to improve ischemic tolerance and protect the aging heart via decreased lipotoxicity, reduced mitochondrial damage, reduced telomere shortening, and preservation of autophagy [41]. In addition, aging-related cardiac proteome remodeling was improved by short-term CR, especially in terms of mitochondrial function, the electron transport train (ETC), and fatty acid metabolism [42]. Our previous studies have demonstrated that an elevation of *Cisd2* levels brings about maintenance of the homeostatic levels of autophagy [43], of cytosolic Ca^2+^ [11,17], and of ROS production, and that this consequently results in the mitochondria that are healthy and well-functioning [11,14].

### 3.2. Comparing the Aging Heart and the Cisd2-Mediated Rejuvenated Heart in Terms of Energy Supply, Protein Transport, and Heart Contraction

Mitochondria play the central role in energy metabolism. The production of ATP by the mitochondria relies on the ETC. In the heart during aging, defects in mitochondrial energy production, impaired mitochondrial dynamics, and deficiencies in the clearance of dysfunctional mitochondria are now recognized as central players that contribute to an aging cardiac phenotype and impaired organ functioning [44]. The main source of increased ROS production during cardiac aging is mitochondrial complex I and complex III [45]. Mitochondrial complex III activity is decreased only in the interfibrillar mitochondria in the aging heart [44]. Two eleven-subunit monomers make up complex III; three of the subunits, namely cytochrome b, cytochrome c1, and the iron–sulfur protein, are involved in electron transfer [44]. *Cisd2* is localized on the mitochondrial outer membrane and endoplasmic reticulum and contains a highly conserved CDGSH (Cys–Asp–Gly–Ser–His) domain; this domain contains the 2Fe–2S of the CisD protein [11,14]. *Cisd2* deficiency results in a decrease in mitochondrial electron transport activity [14]. Thus, a decrease in cardiac *Cisd2* expression during cardiac aging is highly likely to result in increased ROS production and this will consequently lead to elevated oxidative modification of proteins, which will reduce their ability to function [17]. Our previous results proved that a persistently high level of *Cisd2* expression across the whole body throughout an organism’s life is able to ameliorate cardiac aging [17]. In this study, we clearly showed that cardiac-specific overexpression of *Cisd2* during late life can also attenuate cardiac aging. This finding indicates that overexpression of *Cisd2* in the heart has an autonomous effect that prevents cardiac aging.

The other important hallmark of aging is the loss of proteostasis [1,46]. Proteostasis pertains to mechanisms that are involved in the stabilization of correctly folded proteins and the degradation of other proteins, including misfolding by proteasomes or lysosomes [47]. The correct folding of proteins is mediated by chaperones, and their capabilities are known to decrease with age [48]. The derailment of proteostasis involves the impairment of chaperones, changes to the ubiquitin–proteosomal systems, changes to autophagy, and the loss of sarcomeric and cytoskeletal proteins, all of which are related to the induction of cardiomyocyte senescence and thus contribute to cardiac aging [49]. Loss of co-chaperones from the heat shock protein family is known to accelerate aging, whereas activation of HSF-1, the master regulator of the heat shock response and the amyloid-binding components, is known to extend the lifespan [50]. Cardiac aging has been associated with aberrant protein folding, protein aggregate formation, and an increase in protein degradation, all of which are needed to clear toxic misfolded and oxidized proteins. Derailed proteostasis leads to activation of autophagy and various proteases, brings about contractile dysfunction and a disturbance in electric conductance [49,51]. Our transcriptomic results demonstrate that increased protein transport, which occurs as a result of cardiac aging (Figure 4D,E), is reversed by cardiac-specific *Cisd2* overexpression.

### 3.3. Re-Expression of Cisd2 at a Late Age Stage Activates the Sirtuin Pathways, which then Rejuvenate the Aged Myocardium

There are seven sirtuin proteins (SIRT1 to SIRT7) found in mammals, each with a distinct subcellular localization and a relatively precise function. SIRT1, SIRT6, and SIRT7 are mainly located in the nucleus and are involved in the regulation of gene expression through deacetylation. On the other hand, SIRT3, SIRT4, and SIRT5 are largely located in the mitochondria and control various aspects of mitochondrial functioning and metabolism in response to mitochondria stress [52]. Studies have shown that these various sirtuin pathways play the key roles in a number of metabolic and age-related diseases. In terms of cardiovascular disease, the elevation of SIRT1 via either a genetic approach or CR can bring about amelioration of age-dependent cardiac hypertrophy [53,54]. In addition, it has been shown that SIRT7-deficient mice develop cardiomyopathy via an increase in p53 activity [55]. Taking these findings together, it would seem that various sirtuin pathways are a target for the treatment of age-associated cardiac diseases via novel therapeutics. Using transcriptomic analysis, we found that *Cisd2* can rejuvenate the genes involved in various sirtuin pathways (Figure 5A–C). Interestingly, several DEGs are the downstream targets of SIRT3 (namely NDUFA9 and ATP5F1A), SIRT4 (namely PDHA), and SIRT5 (namely GLS). These downstream genes are for the most part involved in the regulation of the tricarboxylic acid cycle, ROS homeostasis, and glutamine metabolism. Additionally, the genes upstream of SIRT1 and downstream of SIRT7 are also rejuvenated in the heart of *Cisd2* icOE mice, which indicates a potential for involvement in the regulation of autophagy and mitochondrion biogenesis. Taken together, overexpression of *Cisd2* in the aging heart would seem to affect activity of a range of sirtuin proteins and their downstream targets resulting in re-orchestration of mitochondrial functions and metabolism.

### 3.4. A Potential Role for Cardiac-Resident Macrophages (CRMs) in the Heart of Cisd2 icOE Mice

Cardiac-resident macrophages (CRMs) are derived from the yolk sac and fetal monocyte progenitor cells; these yolk sac macrophages persist into adulthood [56]. CRMs play an important role in maintaining physiological functions, promoting regeneration, and removing senescent and dying cardiomyocytes. During aging, the embryonically established CRMs can be replaced by infiltrating blood monocytes (Ly6ChiCCR2+) [57]. This turnover of CRMs seems to affect cardiac function, resulting in “inflammaging”, accelerated cardiomyocytes senescence, and cardiac remodeling [58,59]. Intriguingly, our transcriptomic data show that reversal of chemokine signaling and leukocyte extravasation signaling are present in the hearts of *Cisd2* icOE mice. This suggests that in the hearts of *Cisd2* icOE mice, CRMs may be retained as part of the cardiac homeostasis state, whereas the turnover of CRMs that occurs in the naturally aged heart leads to increased numbers of inflammatory macrophages. Furthermore, the phagocytosis pathway was also found to be increased in the aging heart, but this is not the case in the *Cisd2*-rejuvenated heart. This might represent the enhanced clearance of accumulated senescent cardiomyocytes by CRMs during aging. Taken together, “inflammaging” may be returned to homeostasis by CRMs when there is overexpression of *Cisd2* late in life.

### 3.5. Conclusion and Perspectives: Cisd2 as a Potential Molecular Target for Cardiac Rejuvenation

The best way to delay age-associated diseases is to delay or rejuvenate aging itself. The perception is that an anti-aging drug will be able to increase the healthy lifespan; nevertheless, it will not be a fountain of youth nor will it lead to eternal life [60]. In humans, several large meta-analyses of human populations have revealed that antioxidants, folic acid, and B vitamins, as well as multivitamin and mineral supplements, are ineffective in terms of increasing the healthy lifespan. Among the compounds tested by the Interventions Testing Program (ITP), which is directed by the National Institute on Aging, USA, were six compounds, namely aspirin, rapamycin, 17-α-estradiol, acarbose, nordihydroguaiaretic acid, and Protandim, that seemed to be able to significantly extend the lifespan, mainly in male mice [61]. Notably, only rapamycin extended the lifespan in both male and female mice [62]. Other treatments, such as oxaloacetic acid, green tea extract, fish oil, resveratrol, and metformin, did not significantly increase the lifespan according to the ITP studies [63]. However, controversy exists as to whether metformin is able to increase the lifespan of both vertebrates and invertebrates. Interestingly, metformin monotherapy in patients with type 2 diabetes does result in a longer adjusted survival compared with nondiabetic controls [64]. Furthermore, a number of sirtuin-activating compounds have been developed, such as SRT1720 [65] and SRT2104 [66]. These compounds in mice appear to extend both the mean and the maximum lifespan, as well as the healthy lifespan. Furthermore, a previous study revealed that oral administration of a senolytic cocktail, dasatinib plus quercetin, results in selective elimination of senescent cells in naturally aged mice, which then enhances the remaining healthy lifespan and the overall lifespan of old mice [67]. Moreover, naturally derived compounds may also have an indirect effect on cardiovascular health. For example, previous studies revealed that resveratrol, quercetin, epigallocatechin gallate, and curcumin appeared to delay all of the hallmarks of cardiovascular aging and increase the healthy lifespan [68].

**Figure 6:**
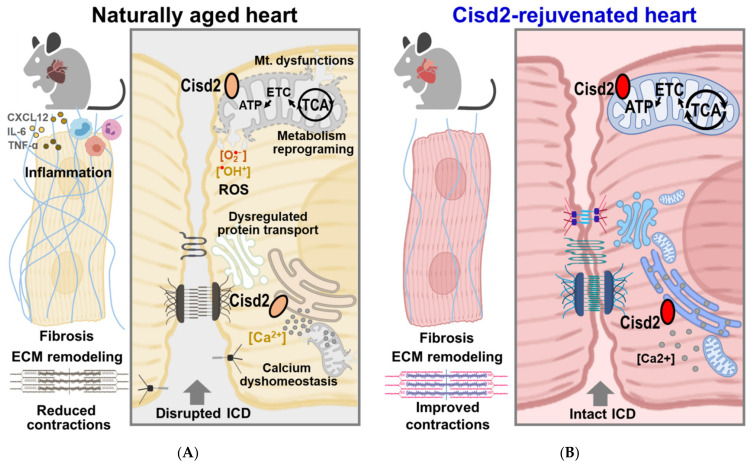
Cardiac-specific overexpression of *Cisd2* at the late-life stage rejuvenates the aging heart. (**A**) In the WT mice at an old age, a decrease in cardiac *Cisd2* results in mitochondrial dysfunction and metabolic reprogramming, as well as in the increase in ROS production, dysregulated proteostasis, and disrupted cytosolic Ca^2+^ homeostasis. Consequently, these defects lead to cellular damage to cardiomyocytes, as well as degeneration of the ICDs, an increase in interstitial fibrosis, and remodeling of the extracellular matrix together with dysregulation of cardiac electromechanical functions. As a result, these functional and structural alterations, which are accompanied by chronic inflammation, bring about cardiac aging. (**B**) In the *Cisd2* icOE mice at an old age, cardiac-specific overexpression of *Cisd2* at the late-life stage appears to rejuvenate the aging heart by reversing the age-related structural damage and the functional decline of the heart. ECM, extracellular matrix; ETC, electron transport chain; ICD, intercalated disc; Mt., mitochondrial; TCA, tricarboxylic acid cycle. The figure was created with BioRender.com.

In WT mice at an old age, a decrease in cardiac *Cisd2* results in mitochondrial dysfunction, metabolic reprogramming, increased ROS production, dysregulation of proteostasis, and disruption of cytosolic Ca^2+^ homeostasis. As a consequence, these defects lead to cellular damage to cardiomyocytes, degeneration of ICDs, an increase in interstitial fibrosis, and remodeling of the extracellular matrix together with dysregulation of electromechanical functioning. These functional and structural alterations are accompanied by chronic inflammation during cardiac aging (Figure 6A). In the *Cisd2* icOE mice at an old age, cardiac-specific overexpression of *Cisd2* at this late-life stage appears to rejuvenate the aging heart via a reversal of the above age-related structural damage and of the disruption to various functions (Figure 6B). Furthermore, we anticipate that in the aging heart, mitochondrion-targeted anti-aging therapy is also brought about by *Cisd2* overexpression at an old age via a restoration of the mitochondrial structure as well as of its functions. Consequently, we suggest that this will be accompanied by an increase in metabolic flexibility, a decrease in ROS production, and preservation of cytosolic Ca^2+^ homeostasis. We believe that all of these changes, taken together, will slow down the aging process in the heart.

## 4. Materials and Methods

### 4.1. Mice

The *ROSA-Cisd2KI/+* mice were generated by introducing *Cisd2* cDNA and a polyA signal downstream to the loxP–STOP–loxP cassette and then knocking-in by homologous recombination at the *ROSA26* locus as described previously [69]. The *αMHC-MerCreMer* mice were purchased from The Jackson Laboratory (JAX stock number 005657; Bar Harbor, ME, USA). The *ROSA-Cisd2KI/+* mice were then crossed with the *αMHC-MerCreMer* mice to generate the *Cisd2* icOE mice (*ROSA-Cisd2KI/+; αMHC-MerCreMerTg/+*). All the mice used in this study were males. All the mice had a pure or congenic *C57BL/6* background. They were bred and housed in a specific pathogen-free (SPF) facility with a 12 h light/12 h dark cycle at a constant temperature (20–22 °C). The animal protocols were approved by the Institutional Animal Care and Use Committee of the Chang Gung Memorial Hospital (Nos. 2017103002, approval date 11 Dec, 2017 and 2017030901, approval date 22 May, 2017) and the National Yang Ming University (No. 1040104r, approval date 26 Sept, 2017). The animal protocol was designed to respect the associated guidelines and the 3R principles (replacement, reduction, and refinement) according to the “Animal Protection Act” of Taiwan.

### 4.2. Cisd2 Induction

Tamoxifen (Sigma-Aldrich, Burlington, MA, USA) was dissolved in ethanol and then further diluted in soybean oil (Sigma-Aldrich) [70]. The mice at the age of 18 months were administered 40 mg/kg body weight on three consecutive days by intraperitoneal injection. The mice were then maintained in an SPF animal facility until sacrifice at 26 months of age.

### 4.3. Western Blotting

Cardiac muscle tissue samples were homogenized using a MagNA Lyser (Roche, Basel, Switzerland) in RIPA buffer and then denatured in a 2% SDS sample. Total protein lysate was separated by SDS-polyacrylamide gel electrophoresis (Bio-Rad, Hercules, CA, USA), which was followed by electro-transfer to a polyvinylidene fluoride transfer membrane (NEF1002001PK, PerkinElmer, Waltham, MA, USA). Next, these membranes were blocked with 5% (*w/v*) non-fat dried milk in TBST for 60 min at room temperature, and then incubated with a primary antibody for 14–16 h at 4 °C. This was followed by washing three times with TBST, probing with the secondary antibody for 60 min at room temperature. Finally, detection was carried by ECL (34580, ThermoFisher Scientific, Waltham, MA, USA). The following antibodies were used for Western blotting: *Cisd2*22 and Gapdh (MAB374, Merk Millipore, Billerica, MA, USA).

### 4.4. Transthoracic Echocardiography

Cardiac function was assessed using a VisualSonics VeVo 2100 Imaging System (VisualSonics, Toronto, ON, Canada). The male mice were anesthetized with 1% isoflurane in 95% O_2_. Body temperature was maintained and monitored at 36–37 °C on a heated pad (TC-1000, CWE Inc., Ardmore, PA, USA). Cardiac function was assessed using a high-frequency 30–50 MHz probe. Data analysis was carried out using the VisualSonics software (VisualSonics, Toronto, ON, Canada). The personnel responsible for data acquisition were blinded to the animal groupings.

### 4.5. Electrocardiography (ECG)

Functional testing of the cardiac ECGs of all the mice was performed as described previously [17]. All the procedures took place during the light phase. Anesthesia was initially induced by placing the mice for 3-5 min in a chamber filled with 3% volume-to-volume isoflurane. The mice were then positioned on a warm pad for the duration of the ECG recording. Anesthesia was maintained via inhalation of 1.5% isoflurane through a nose cone. Continuous 5-min ECGs were obtained using subcutaneous electrodes and recorded using a PowerLab data acquisition system (model ML866, ADInstruments, Dunedin, New Zealand) and an Animal Bio Amp (model ML136, ADInstruments; Dunedin, New Zealand). ECG analysis was performed in an unbiased fashion using LabChart 7 Pro version 7.3.1 (ADInstruments, Inc., Dunedin, New Zealand). Detection and analysis of the QTc intervals, QRS intervals, and Tpeak-Tend intervals were set to the Mouse ECG parameters. The values obtained were compared statistically using the Mann-Whitney U test, and a *p*-value < 0.05 was accepted as significant.

### 4.6. Immunofluorescence (IF), Confocal Microscopy, and Cx43/Pan-Cadherin Co-Localization Coefficiency

Immunofluorescent staining was performed as previously described [17]. Briefly, optimal cutting temperature embedded cryosections (16 μm) were stained using the following antibodies: pan-cadherin (C3678, Sigma-Aldrich, Billerica, MA, USA), Desmoplakin (CBL173, Merk Millipore, Billerica, MA, USA), Cx43 (C8093, Sigma-Aldrich, Billerica, MA, USA), Collagen I (ab270993, Abcam, Cambridge, UK), and α-actinin (A7811, Sigma-Aldrich, Billerica, MA, USA), wheat germ agglutinin, or α-actinin (rhodamine phalloidin) was co-stained with nucleus staining dye DAPI or Hoechst. Gap junction remodeling was quantified by determining the extent of co-localization of Cnx43 and pan-cadherin at the intercalated discs using the Pearson–Spearman correlation co-localization (PSC) plugin for ImageJ (National Institutes of Health, Bethesda, MD, USA).

### 4.7. RNA Isolation from Tissue, RNA Sequencing, and Pathway Analysis

Total RNA was isolated from the cardiac muscle of the left ventricle using TRI Reagent (T9424, Sigma) and phenol/chloroform extraction. The quality of the total RNA was assessed using an Agilent 2100 Bioanalyzer (Agilent Technologies, Santa Clara, CA, USA); the samples with an RNA integrity number (RIN) higher than 8 were subjected to RNA sequencing. RNA sequencing (RNA-seq) was conducted at the Genome Research Center at the National Yang Ming Chiao Tung University. The analysis was generated to a depth of at least 20 million reads for each sample by single-end sequencing. After mapping, the unique gene reads were analyzed as RPKM (reads per kilobase of exon model per million reads) to assess gene expression. A total of 5686 genes were retained after filtering in order to identify expressed genes in the heart (minimal counts in RPKM > 4 detected in at least 50% of the samples). The *p*-values of the genes, which were identified using unpaired Student’s *t*-tests, were adjusted using the Benjamini–Hochberg method. Differentially expressed genes (DEGs) were identified using a false discovery rate (FDR) cutoff threshold as indicated in the figure legends. DEGs reversed by cardiac-specific induction of *CISD2* overexpression (*Cisd2* icOE) were analyzed using the following criteria: (1) 26-mo WT vs. 3-mo WT, FDR < 0.25; (2) 26-mo WT vs. 26-mo *Cisd2* icOE, *p* < 0.05, and by a reversal of the 26-mo WT-Veh vs. 3-mo WT. Gene Ontology (GO) functional characterization was performed using the online tool DAVID Functional Annotation Bioinformatics Microarray Analysis (https://david.ncifcrf.gov, accessed 30 August 2021). Pathway analysis of the DEGs was carried out by QIAGEN ingenuity pathway analysis (IPA) (pathway *p* < 0.05). Heatmaps were created by loading the log-transformed fold changes into the MultiExperiment Viewer 4.9 software.

### 4.8. Statistical Analysis

The data are presented as the means ± SD as described in the figure legends. Comparisons between groups of more than two were carried out using one-way ANOVA with the Bonferroni multiple comparison test as indicated in the figure legends. When analyzing statistical differences between the groups, a *p*-value of < 0.05 was considered significant. Statistical analysis was carried out using the software package Graphpad Prism 6.0.

## Data Availability

Due to ethical restrictions, the data presented in this study are available on request from the corresponding author.

## Supplementary Materials

The following are available online at https://www.mdpi.com/article/10.3390/ijms222111487/s1.

## References

[1] Lopez-Otin C., Blasco M.A., Partridge L., Serrano M., Kroemer G. (2013). The hallmarks of aging. Cell.

[2] Picca A., Mankowski R.T., Burman J.L., Donisi L., Kim J.-S., Marzetti E., Leeuwenburgh C. (2018). Mitochondrial quality control mechanisms as molecular targets in cardiac ageing. Nat. Rev. Cardiol..

[3] Benjamin E.J., Muntner P., Alonso A., Bittencourt M.S., Callaway C.W., Carson A.P., Chamberlain A.M., Chang A.R., Cheng S., Das S.R. (2019). Heart Disease and Stroke Statistics-2019 Update: A Report From the American Heart Association. Circulation.

[4] Danaei G., Ding E.L., Mozaffarian D., Taylor B., Rehm J., Murray C.J.L., Ezzati M. (2009). The preventable causes of death in the United States: Comparative risk assessment of dietary, lifestyle, and metabolic risk factors. PLoS Med..

[5] Kubli D.A., Sussman M.A. (2017). Editorial commentary: Mitochondrial autophagy in cardiac aging is all fluxed up. Trends Cardiovasc. Med..

[6] Baker D.J., Childs B.G., Durik M., Wijers M.E., Sieben C.J., Zhong J., Saltness R.A., Jeganathan K., Verzosa G.C., Pezeshki A.-M. (2016). Naturally occurring p16Ink4a-positive cells shorten healthy lifespan. Nat. Cell Biol..

[7] Korski K.I., Kubli D.A., Wang B.J., Khalafalla F.G., Monsanto M.M., Firouzi F., Echeagaray O.H., Kim T., Adamson R.M., Dembitsky W.P. (2019). Hypoxia Prevents Mitochondrial Dysfunction and Senescence in Human c-Kit+ Cardiac Progenitor Cells. Stem Cells.

[8] Rodríguez M.I., Carretero M., Escames G., Lopez L.C., Maldonado M.D., Tan D.-X., Reiter R.J., Acuña-Castroviejo D. (2007). Chronic melatonin treatment prevents age-dependent cardiac mitochondrial dysfunction in senescence-accelerated mice. Free. Radic. Res..

[9] Häseli S., Deubel S., Jung T., Grune T., Ott C. (2020). Cardiomyocyte Contractility and Autophagy in a Premature Senescence Model of Cardiac Aging. Oxidative Med. Cell. Longev..

[10] Lin B., Feng D., Xu J. (2019). Cardioprotective effects of microRNA-18a on acute myocardial infarction by promoting cardiomy-ocyte autophagy and suppressing cellular senescence via brain derived neurotrophic factor. Cell Biosci..

[11] Yeh C.-H., Chou Y.-J., Kao C.-H., Tsai T.-F. (2020). Mitochondria and Calcium Homeostasis: *Cisd2* as a Big Player in Cardiac Ageing. Int. J. Mol. Sci..

[12] Shi R., Guberman M., Kirshenbaum L.A. (2018). Mitochondrial quality control: The role of mitophagy in aging. Trends Cardiovasc. Med..

[13] Dutta D., Calvani R., Bernabei R., Leeuwenburgh C., Marzetti E. (2012). Contribution of impaired mitochondrial autophagy to cardiac aging: Mechanisms and therapeutic opportunities. Circ. Res..

[14] Shen Z.-Q., Huang Y.-L., Teng Y.-C., Wang T.-W., Kao C.-H., Yeh C.-H., Tsai T.-F. (2021). *CISD2* maintains cellular homeostasis. Biochim. et Biophys. Acta Bioenerg..

[15] Chen Y.-T., Kao C.-H., Wang C.-H., Wu C.-Y., Tsai C.-Y., Liu F.-C., Yang C.-W., Wei Y.-H., Hsu M.-T., Tsai S.-F. (2009). *Cisd2* deficiency drives premature aging and causes mitochondria-mediated defects in mice. Genes Dev..

[16] Shen Z.-Q., Chen Y.-F., Chen J.-R., Jou Y.-S., Wu P.-C., Kao C.-H., Wang C.-H., Huang Y.-L., Chen C.-F., Huang T.-S. (2017). *CISD2* Haploinsufficiency Disrupts Calcium Homeostasis, Causes Nonalcoholic Fatty Liver Disease, and Promotes Hepatocellular Carcinoma. Cell Rep..

[17] Yeh C.-H., Shen Z.-Q., Hsiung S.-Y., Wu P.-C., Teng Y.-C., Chou Y.-J., Fang S.-W., Chen C.-F., Yan Y.-T., Kao L.-S. (2019). *Cisd2* is essential to delaying cardiac aging and to maintaining heart functions. PLoS Biol..

[18] Chen Y.-F., Kao C.-H., Kirby R., Tsai T.-F. (2009). *Cisd2* mediates mitochondrial integrity and life span in mammals. Autophagy.

[19] Wang C.-H., Chen Y.-F., Wu C.-Y., Wu P.-C., Huang Y.-L., Kao C.-H., Lin C.-H., Kao L.-S., Tsai T.-F., Wei Y.-H. (2014). *Cisd2* modulates the differentiation and functioning of adipocytes by regulating intracellular Ca^2+^ homeostasis. Hum. Mol. Genet..

[20] Huang Y.L., Shen Z.Q., Wu C.Y., Teng Y.C., Liao C.C., Kao C.H., Chen L.K., Lin C.H., Tsai T.F. (2018). Comparative pro-teomic profiling reveals a role for *Cisd2* in skeletal muscle aging. Aging Cell.

[21] Chen Y.F., Chou T.Y., Lin I.H., Chen C.G., Kao C.H., Huang G.J., Chen L.K., Wang P.N., Lin C.P., Tsai T.F. (2020). Upreg-ulation of *Cisd2* attenuates Alzheimer’s-related neuronal loss in mice. J. Pathol..

[22] Wu C.-Y., Chen Y.-F., Wang C.-H., Kao C.-H., Zhuang H.-W., Chen C.-C., Chen L.-K., Kirby R., Wei Y.-H., Tsai S.-F. (2012). A persistent level of *Cisd2* extends healthy lifespan and delays aging in mice. Hum. Mol. Genet..

[23] Sohal D.S., Nghiem M., Crackower M.A., Witt S.A., Kimball T.R., Tymitz K.M., Penninger J.M., Molkentin J.D. (2001). Tem-porally regulated and tissue-specific gene manipulations in the adult and embryonic heart using a tamoxifen-inducible Cre protein. Circ. Res..

[24] Peng F., Xie F., Muzik O. (2018). Alteration of Copper Fluxes in Brain Aging: A Longitudinal Study in Rodent Using 64CuCl_2_-PET/CT. Aging Dis..

[25] Strait J.B., Lakatta E.G. (2012). Aging-Associated Cardiovascular Changes and Their Relationship to Heart Failure. Heart Fail. Clin..

[26] Nagibin V., Egan Benova T., Viczenczova C., Szeiffova Bacova B., Dovinova I., Barancik M., Tribulova N. (2016). Ageing related down-regulation of myocardial connexin-43 and up-regulation of MMP-2 may predict propensity to atrial fibrillation in ex-perimental animals. Physiol. Res..

[27] Wright K.J., Thomas M.M., Betik A.C., Belke D., Hepple R.T. (2014). Exercise training initiated in late middle age attenuates cardiac fibrosis and advanced glycation end-product accumulation in senescent rats. Exp. Gerontol..

[28] Teng Y.-C., Wang J.-Y., Chi Y.-H., Tsai T.-F. (2020). Exercise and the *Cisd2* Prolongevity Gene: Two Promising Strategies to Delay the Aging of Skeletal Muscle. Int. J. Mol. Sci..

[29] Banks L., Buchan T.A., Dizonno V. (2016). Aerobic exercise attenuates ageing of the athletic heart. J. Physiol..

[30] Vagnozzi R.J., Kasam R.K., Sargent M.A., Molkentin J.D. (2021). Cardiac Cell Therapy Fails to Rejuvenate the Chronically Scarred Rodent Heart. Circulation.

[31] Bolli R. (2020). Cell therapy for acute myocardial infarction: Requiescat in Pace. Eur. Heart J..

[32] Rafatian G., Kamkar M., Parent S., Michie C., Risha Y., Molgat A.S.D., Seymour R., Suuronen E.J., Davis D.R. (2020). Mybl2 rejuvenates heart explant-derived cells from aged donors after myocardial infarction. Aging Cell.

[33] Travers J.G., Kamal F.A., Robbins J., Yutzey K.E., Blaxall B.C. (2016). Cardiac Fibrosis: The fibroblast awakens. Circ. Res..

[34] Aghajanian H., Kimura T., Rurik J.G., Hancock A.S., Leibowitz M.S., Li L., Scholler J., Monslow J., Lo A., Han W. (2019). Targeting cardiac fibrosis with engineered T cells. Nature.

[35] Baker D.J., Wijshake T., Tchkonia T., LeBrasseur N., Childs B.G., Van De Sluis B., Kirkland J.L., Van Deursen J.M. (2011). Clearance of p16Ink4a-positive senescent cells delays ageing-associated disorders. Nature.

[36] Su Y.-J., Wang P.-W., Weng S.-W. (2021). The Role of Mitochondria in Immune-Cell-Mediated Tissue Regeneration and Ageing. Int. J. Mol. Sci..

[37] Weixler V., Lapusca R., Grangl G., Guariento A., Saeed M.Y., Cowan D.B., Del Nido P.J., McCully J.D., Friehs I. (2021). Au-togenous mitochondria transplantation for treatment of right heart failure. J. Thorac. Cardiovasc. Surg..

[38] Mietsch M., Hinkel R. (2021). "Empowering" Cardiac Cells via Stem Cell Derived Mitochondrial Transplantation- Does Age Matter?. Int. J. Mol. Sci..

[39] Liang W.J., Gustafsson B. (2020). The Aging Heart: Mitophagy at the Center of Rejuvenation. Front. Cardiovasc. Med..

[40] Li X.-D., Rebrin I., Forster M.J., Sohal R.S. (2012). Effects of age and caloric restriction on mitochondrial protein oxidative damage in mice. Mech. Ageing Dev..

[41] Sheng Y., Lv S., Huang M., Lv Y., Yu J., Liu J., Tang T., Qi H., Di W., Ding G. (2017). Opposing effects on cardiac function by calorie restriction in different-aged mice. Aging Cell.

[42] Dai D.-F., Karunadharma P.P., Chiao Y.A., Basisty N., Crispin D., Hsieh E.J., Chen T., Gu H., Djukovic D., Raftery D. (2014). Altered proteome turnover and remodeling by short-term caloric restriction or rapamycin rejuvenate the aging heart. Aging Cell.

[43] Wang C.H., Kao C.H., Chen Y.F., Wei Y.H., Tsai T.F. (2014). *Cisd2* mediates lifespan: Is there an interconnection among Ca(2)(+) homeostasis, autophagy, and lifespan?. Free Radic Res..

[44] Lesnefsky E.J., Chen Q., Hoppel C.L. (2016). Mitochondrial Metabolism in Aging Heart. Circ. Res..

[45] Kuka S., Tatarkova Z., Račay P., Lehotský J., Dobrota D., Kaplan P. (2014). Effect of aging on formation of reactive oxygen species by mitochondria of rat heart. Gen. Physiol. Biophys..

[46] Martinez-Miguel V.E., Lujan C., Espie-Caullet T., Martinez-Martinez D., Moore S., Backes C., Gonzalez S., Galimov E.R., Brown A.E.X., Halic M. (2021). Increased fidelity of protein syn-thesis extends lifespan. Cell Metab..

[47] Koga H., Kaushik S., Cuervo A.M. (2011). Protein homeostasis and aging: The importance of exquisite quality control. Ageing Res. Rev..

[48] Anisimova A., Alexandrov A.I., Makarova N.E., Gladyshev V.N., Dmitriev S.E. (2018). Protein synthesis and quality control in aging. Aging.

[49] Henning R., Brundel B.J.J.M. (2017). Proteostasis in cardiac health and disease. Nat. Rev. Cardiol..

[50] Alavez S., Vantipalli M.C., Zucker D.J., Klang I.M., Lithgow G.J. (2011). Amyloid-binding compounds maintain protein home-ostasis during ageing and extend lifespan. Nature.

[51] Zhang D., Hu X., Henning R.H., Brundel B.J. (2016). Keeping up the balance: Role of HDACs in cardiac proteostasis and ther-apeutic implications for atrial fibrillation. Cardiovasc. Res..

[52] Lee I.H. (2019). Mechanisms and disease implications of sirtuin-mediated autophagic regulation. Exp. Mol. Med..

[53] Alcendor R.R., Gao S., Zhai P., Zablocki D., Holle E., Yu X., Tian B., Wagner T., Vatner S.F., Sadoshima J. (2007). Sirt1 regu-lates aging and resistance to oxidative stress in the heart. Circ. Res..

[54] Lavu S., Boss O., Elliott P.J., Lambert P.D. (2008). Sirtuins—novel therapeutic targets to treat age-associated diseases. Nat. Rev. Drug Discov..

[55] Vakhrusheva O., Smolka C., Gajawada P., Kostin S., Boettger T., Kubin T., Braun T., Bober E. (2008). Sirt7 Increases Stress Resistance of Cardiomyocytes and Prevents Apoptosis and Inflammatory Cardiomyopathy in Mice. Circ. Res..

[56] Epelman S., Lavine K.J., Beaudin A.E., Sojka D.K., Carrero J.A., Calderon B., Brija T., Gautier E., Ivanov S., Satpathy A. (2014). Embryonic and Adult-Derived Resident Cardiac Macrophages Are Maintained through Distinct Mechanisms at Steady State and during Inflammation. Immunity.

[57] Zhang S., Chen R., Chakrabarti S., Su Z. (2020). Resident macrophages as potential therapeutic targets for cardiac ageing and injury. Clin. Transl. Immunol..

[58] Wagner J.U., Dimmeler S. (2019). Cellular cross-talks in the diseased and aging heart. J. Mol. Cell. Cardiol..

[59] Meschiari C.A., Ero O.K., Pan H., Finkel T., Lindsey M.L. (2017). The impact of aging on cardiac extracellular matrix. GeroScience.

[60] Hayden E.C. (2015). Anti-ageing pill pushed as bona fide drug. Nat. Cell Biol..

[61] Nadon N.L., Strong R., Miller R.A., Harrison D.E. (2016). NIA Interventions Testing Program: Investigating Putative Aging Intervention Agents in a Genetically Heterogeneous Mouse Model. EBioMedicine.

[62] Harrison D.E., Strong R., Sharp Z.D., Nelson J.F., Astle C.M., Flurkey K., Nadon N.L., Wilkinson J.E., Frenkel K., Carter C.S. (2009). Rapamycin fed late in life extends lifespan in genetically heterogeneous mice. Nature.

[63] Strong R., Miller R.A., Antebi A., Astle C.M., Bogue M., Denzel M., Fernandez E., Flurkey K., Hamilton K.L., Lamming D.W. (2016). Longer lifespan in male mice treated with a weakly estrogenic agonist, an antioxidant, an α-glucosidase inhibitor or a Nrf2-inducer. Aging Cell.

[64] Bannister C., Holden S.E., Jenkins-Jones S., Morgan C.L., Halcox J., Schernthaner G., Mukherjee J., Currie C.J. (2014). Can people with type 2 diabetes live longer than those without? A comparison of mortality in people initiated with metformin or sulphonylurea monotherapy and matched, non-diabetic controls. Diabetes, Obes. Metab..

[65] Minor R.K., Baur J., Gomes A.P., Ward T.M., Csiszar A., Mercken E.M., Abdelmohsen K., Shin Y.-K., Canto C., Scheibye-Knudsen M. (2011). SRT1720 improves survival and healthspan of obese mice. Sci. Rep..

[66] Mercken E.M., Mitchell S.J., Martin-Montalvo A., Minor R.K., Almeida M., Gomes A.P., Scheibye-Knudsen M., Palacios H.H., Licata J.J., Zhang Y. (2014). SRT 2104 extends survival of male mice on a standard diet and preserves bone and muscle mass. Aging Cell.

[67] Xu M., Pirtskhalava T., Farr J.N., Weigand B.M., Palmer A.K., Weivoda M.M., Inman C.L., Ogrodnik M., Hachfeld C.M., Fraser D.G. (2018). Senolytics improve physical function and increase lifespan in old age. Nat. Med..

[68] Khurana S., Venkataraman K., Hollingsworth A., Piche M., Tai T.C. (2013). Polyphenols: Benefits to the Cardiovascular System in Health and in Aging. Nutrients.

[69] Soriano P. (1999). Generalized lacZ expression with the ROSA26 Cre reporter strain. Nat. Genet..

[70] Nakamura E., Nguyen M.T., Mackem S. (2006). Kinetics of tamoxifen-regulated Cre activity in mice using a cartilage-specific CreER(T) to assay temporal activity windows along the proximodistal limb skeleton. Dev. Dyn..

