# Supplementary material for: Rejuvenating the Aging Heart by Enhancing the Expression of the Cisd2 Prolongevity Gene
Source: Int J Mol Sci. 2021 Oct 25;22(21):11487. doi: 10.3390/ijms222111487 (PMC8583758; doi:10.3390/ijms222111487)
Supplement: Supplementary file 1 [file ijms-22-11487-s001.zip › ijms-1433039-supplementary.pdf]

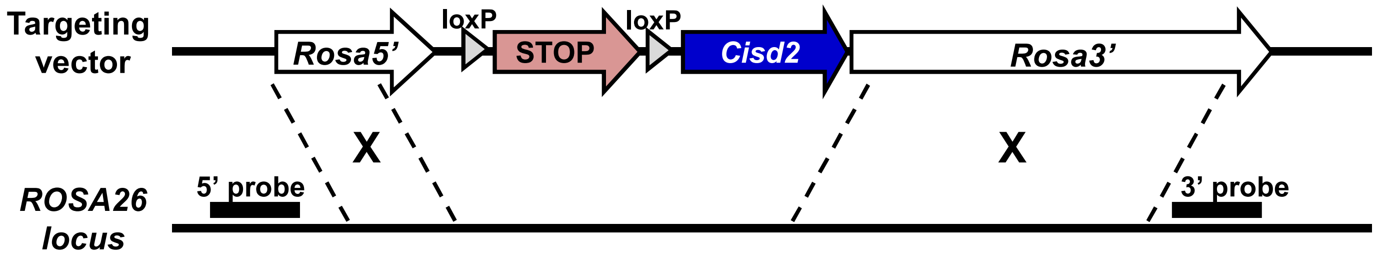

Supplementary Figure S1. Strategy for generating ROSA26-Cisd2KI/+ mice.
Schematic diagram depicting the Cisd2 targeting vector that targets the ROSA26 genomic locus after homologous recombination. cDNA sequence encoding Cisd2 protein was cloned into the targeting vector behind a STOP cassette which was flanked by two loxP sites.

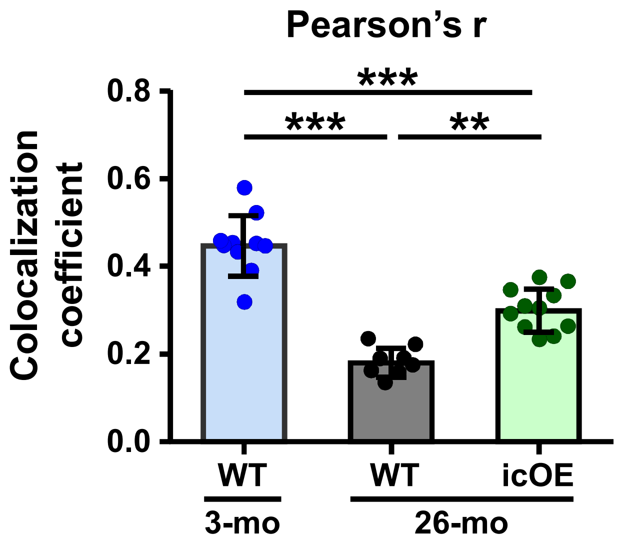

Supplementary Figure S2. Colocalization coefficient of gap junction protein. Colocalization coefficient of gap junction protein (Cx43) and intercalated disc protein (pan-cadherin) was analyzed by Pearson's Correlation. The computed value is presented as the Cx43/pan-cadherin colocalization coefficient. Data were collected from five randomly selected fields for each heart sample. Data are presented as mean ± SD and are analyzed by one way ANOVA with Bonferroni multiple comparison test. ***p*<0.005; ****p*<0.001. Mouse number n≥4 for each group.
